# Amperometric inhibitive biosensor based on horseradish peroxidase-nanoporous gold for sulfide determination

**DOI:** 10.1038/srep30905

**Published:** 2016-08-12

**Authors:** Huihui Sun, Zhuang Liu, Chao Wu, Ping Xu, Xia Wang

**Affiliations:** 1 State Key Laboratory of Microbial Technology, Shandong University, Jinan 250100, P. R. China

## Abstract

As a well-known toxic pollutant, sulfide is harmful to human health. In this study, a simple and sensitive amperometric inhibitive biosensor was developed for the determination of sulfide in the environment. By immobilizing nanoporous gold (NPG) on glassy carbon electrode (GCE), and encapsulating horseradish peroxidase (HRP) onto NPG, a HRP/NPG/GCE bioelectrode for sulfide detection was successfully constructed based on the inhibition of sulfide on HRP activity with *o*-Phenylenediamine (OPD) as a substrate. The resulted HRP/NPG/GCE bioelectrode achieved a wide linear range of 0.1–40 μM in sulfide detection with a high sensitivity of 1720 μA mM^−1^ cm^−2^ and a low detection limit of 0.027 μM. Additionally, the inhibition of sulfide on HRP is competitive inhibition with OPD as a substrate by Michaelis-Menten analysis. Notably, the recovery of HRP activity was quickly achieved by washing the HRP/NPG/GCE bioelectrode using differential pulse voltammetry (DPV) technique in deaerated PBS (50 mM, pH 7.0) for only 60 s. Furthermore, the real sample analysis of sulfide by the HRP/NPG/GCE bioelectrode was achieved. Based on above results, the HRP/NPG/GCE bioelectrode could be a better choice for the real determination of sulfide compared to inhibitive biosensors previously reported.

Sulfide, which represents the sum of H_2_S, HS^−^, S^2−^, can be widely found in the environment especially in natural water and wastewater^1^. While, as a well-known toxic pollutant, sulfide could cause serious environmental issues that threatening human health^2^. Thus, sensitive and simple method to detect sulfide in the environment is necessary for monitoring and controlling its toxicity. A variety of methods for determining sulfide have been reported, such as spetrophotometry^3^,^4^, chemiluminescence^5^, chromatography^6^, and fluorescence^7^,^8^,^9^. However, these traditional methods possess many limitations such as consumed time, complex sample pretreatment, high cost, low sensitivity, and secondary pollution. Therefore, it is urgent to develop a new method for the simple and effective determination of sulfide.

Compared with traditional methods, biosensor has prominent advantages such as simple sample pretreatment, high selectivity and sensitivity, easy operation, and quick response^1^,^10^. Among that, biosensors based on the inhibition towards enzyme activity by sulfide were widely reported for sulfide detection^1^,^2^,^11^,^12^. The enzymes commonly used in inhibitive biosensors for sulfide detection were horseradish peroxidase (HRP)^1^,^2^,^11^, fungal peroxidase^12^, cytochrome C oxidase^13^, and ascorbate oxidase^14^. Yang *et al*.^11^ developed a HRP inhibitive biosensor using immobilized HRP on Au electrode by cross-linked with glutaraldehyde. Savizi *et al*.^12^ constructed an inhibitive biosensor with immobilized *Coprinus cinereus* peroxidase on screen printed electrode using chitosan and acrylamide as binders. However, these biosensors had narrow detection ranges (0.5–12.7 μM and 1.09–16.3 μM, respectively) with complex preparing and handling processes. Especially, the enzyme binder used in bioelectrode fabrication might block the electron transfer and the recovery of enzyme activity. In spite of Liu *et al*.^1^ and Shan *et al*.^2^ improved the detecting range of sulfide, their biosensors showed poor anti-interference ability and low sensitivity, respectively. Therefore, developing a binder-free inhibitive biosensor with simple fabrication process, high sensitivity, and wide detection range is still a challenge for sulfide determination.

In our previous work, HRP was successfully assembled onto nanoporous gold (NPG) to form a HRP/NPG biocomposite^15^, and then the HRP/NPG biocomposite was immobilized on glassy carbon electrode (GCE) to construct a HRP/NPG/GCE bioelectrode. The resulted HRP/NPG/GCE bioelectrode achieved good performances in detecting the mixture of phenols and aromatic amines^16^. Further, efficient direct electron transfer between HRP molecule and electrode was achieved due to the unique physical and chemical properties of NPG^16^. Compared to traditional materials, NPG was demonstrated to be an excellent binder-free enzyme carrier due to its reproducible three-dimensional porous structure, high surface area, good biocompatibility, and unique electrochemical catalytic performance^17^,^18^. Especially, NPG has good conductivity, which is critical for efficient electron transfer between enzyme and electrode^19^. Hence, the HRP/NPG/GCE bioelectrode could achieve reproducible enzyme activity and efficient electron transfer with simple operation. Considering the excellent sensing performance and easy recovery of HRP activity, the HRP/NPG/GCE bioelectrode could be efficient in constructing inhibitive sulfide biosensor.

In this work, the HRP/NPG/GCE bioelectrode was further studied in sulfide detection based on our previous research and the inhibitive effect towards HRP by sulfide. *o*-Phenylenediamine (OPD) was selected as a substrate. The HRP/NPG/GCE bioelectrode was expected to exhibit high sensitivity, wide detection range, and selectivity for sulfide determination.

## Results and Discussion

**Figure 1:**
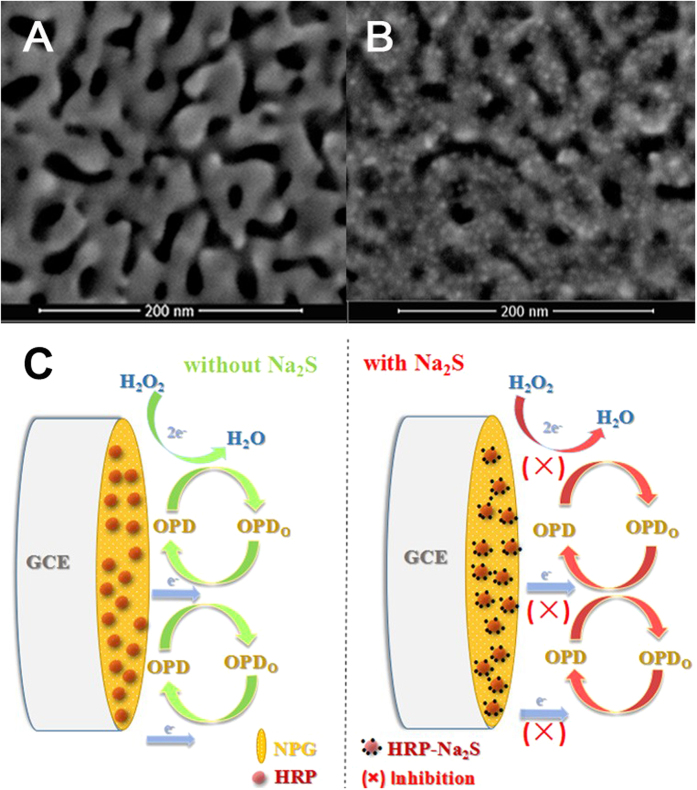
SEM images of NPG with a pore size of 35 nm before (**A**) and after HRP loading (**B**); Electrochemical reaction of HRP/NPG/GCE bioelectrode (**C**).

The morphology of NPG/GCE electrode (Fig. 1A) and HRP/NPG/GCE bioelectrode (Fig. 1B) were characterized by scanning electron microscope (SEM). Before HRP loading, an open three-dimensional nanoporous structure of NPG was observed, which provide an excellent electrochemical reaction interface to link electrode and enzyme^20^,^21^,^22^. Compared to bare NPG, a coarser surface morphology and smaller pore size of NPG (Fig. 1B) were observed for the HRP/NPG/GCE bioelectrode, confirming that HRP was immobilized over the ligament site of NPG with high radial curvatures^16^. These results demonstrated that NPG was an excellent binder-free enzyme carrier with good biocompatibility.

**Figure 2:**
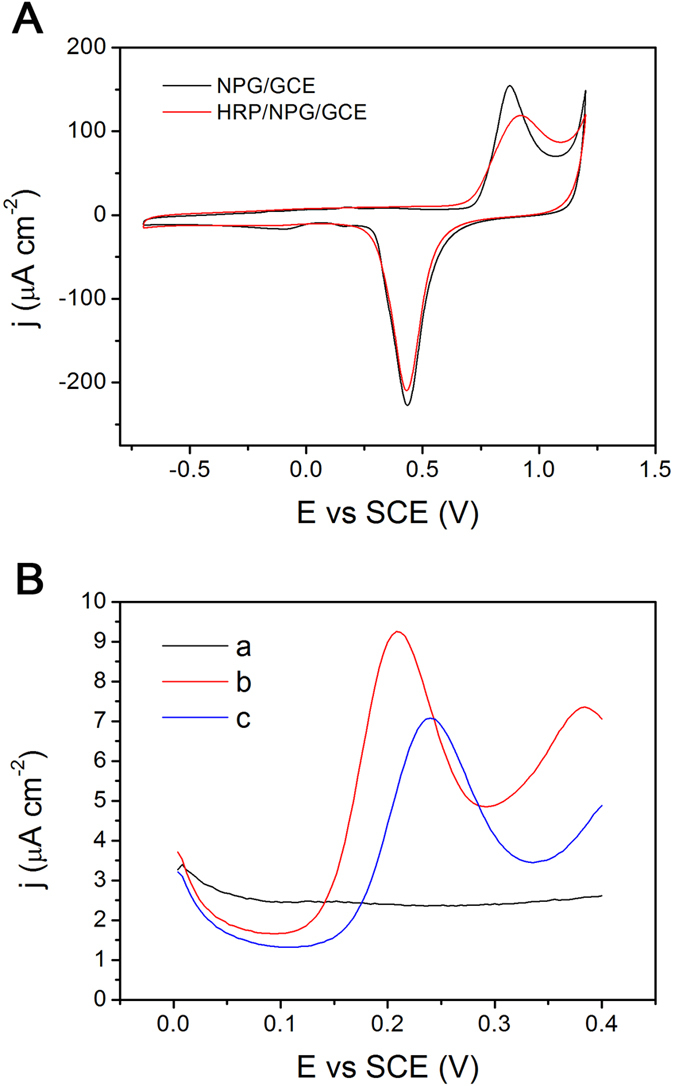
(**A**) CVs of NPG/GCE electrode and HRP/NPG/GCE bioelectrode in deaerated PBS (50 mM, pH 7.0); (**B**) DPVs of HRP/NPG/GCE bioelectrode at different conditions: (a) in deaerated PBS (50 mM, pH 7.0); (b) the same as (a) with the presence of 100 μM OPD and 0.1 mM H_2_O_2_; (c) the same as (b) with 10 μM sulfide.

The electrochemical determination of the NPG/GCE electrode and the HRP/NPG/GCE bioelectrode were recorded in deaerated PBS (50 mM, pH 7.0) using cyclic voltammetry (CV) technique at a scan rate of 50 mV s^−1^, respectively. Obviously, the peak current density of the HRP/NPG/GCE bioelectrode was lower than that of the NPG/GCE electrode as shown in Fig. 2A. The reason is that the HRP immobilized on NPG behaved as a barrier for electron transfer, which led to a lower current response. These results also proved the successful immobilization of HRP on NPG^20^,^23^.

To confirm the function of the HRP/NPG/GCE bioelectrode during sulfide detection, the differential pulse voltammetry (DPV) curves of the HRP/NPG/GCE bioelectrode were measured between +0.0 V and +0.4 V (Fig. 2B). Figure 2B-a showed the DPV curve of the HRP/NPG/GCE bioelectrode in deaerated PBS (50 mM, pH 7.0). Notably, with the presence of 100 μM OPD accompanied with 0.1 mM H_2_O_2_, an obvious, rapid, and stable oxidation peek current response of the HRP/NPG/GCE bioelectrode was observed at +0.21 V (Fig. 2B-b). In contrast, when 10 μM sulfide was added, a remarkably decreased peak current density was observed as shown in Fig. 2B-c, confirming the inhibition of sulfide on HRP activity. These results indicated that the inhibitive biosensor construction was feasible using the HRP/NPG/GCE bioelectrode.

Commonly, HRP contains two different types of metal center, iron (III) protoporphyrin IX and two calcium atoms. Further, substrate oxidation by HRP occurs at the ‘exposed’ heme edge of iron (III) protoporphyrin IX. The working mechanism of the HRP/NPG/GCE bioelectrode during the electrocatalytic oxidation of OPD could be expressed as follows, which was modified based on Ruzgas^24^ and Yang^11^:


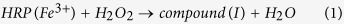



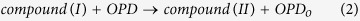



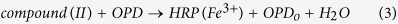



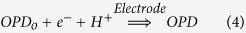


Among these reactions, HRP (Fe^3+^) was oxidized by hydrogen peroxide to compound I. Then compound I was reduced by OPD to compound II. Then compound II was reduced by redox mediator OPD to its original form of HRP (Fe^3+^), and OPD was oxidized to OPD_O_ in both reaction (2) and (3). OPD_O_ was subsequently reduced back to OPD by electrode as described in Equation (4). However, when sulfide was added into the sample solution, it could attack the heme group of HRP^1^ and inhibit the transmission of electrons as shown in Fig. 1C. Therefore, sulfide could inhibit the activity of HRP that resulting in a decrease of current.

To confirm the type of inhibition between HRP and sulfide, the analysis of enzyme-substrate kinetics was studied. The electrocatalytic reaction of substrate can be expressed as Lineweaver-Burk equation^25^:


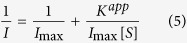


where *I* represents the current at different substrate concentration, *I*_*max*_ is the saturation value of the current. [S] is the substrate concentration and *K*^*app*^ is the apparent Michaelis-Menten constant. The *I*_*max*_ and *K*^*app*^ values with the absence and presence of 2 μM sulfide were calculated respectively, as shown in Table 1. When H_2_O_2_ served as a substrate, values for *I*_*max*_ and *K*^*app*^ with sulfide were all smaller than that without inhibitor, indicating that the sulfide inhibition on HRP was uncompetitive inhibition with H_2_O_2_. This result was consistent with previous study that the type of inhibition between sulfide and HRP was uncompetitive inhibition with H_2_O_2_ as a substrate^11^. When the substrate was OPD, *I*_max_ and *K*^app^ values with the absence and presence of 2 μM sulfide were also calculated, respectively. As shown in Table 1, a similar *I*_*max*_ and higher *K*^*app*^ with sulfide were obtained compared to that without inhibitor, which indicated that the inhibition on HRP by sulfide was competitive inhibition with OPD. Therefore, sulfide and OPD would competitively occupy the active sites of HRP during electrochemical reaction. As an inhibitive biosensor, the detection of sulfide was achieved by decreasing current response caused by the sulfide inhibition on HRP. During the competitive inhibitive process, sulfide would replace OPD on active sites of HRP. Since the original current response was based on the electrocatalysis of OPD on the active sites of HRP, the replacement of OPD by sulfide could result in a more evident current decrease than that of uncompetitive inhibition. As a result, the competitive inhibition towards HRP with OPD as a substrate could lead to a higher sensitivity in sulfide detection than the uncompetitive inhibition with H_2_O_2_ as a substrate. This was further confirmed by the lower *K*^app^ and higher *I*_max_ as shown in Table 1. Hence, the determination of sulfide was conducted using the HRP/NPG/GCE bioelectrode with OPD as a substrate.

**Figure 3:**
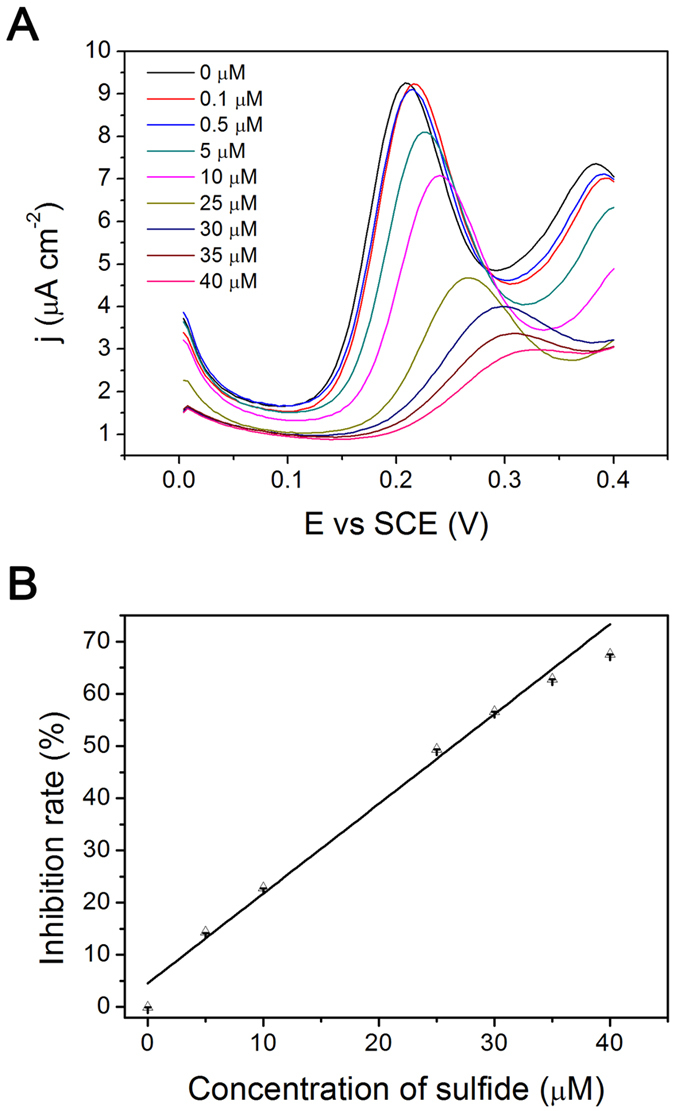
(**A**) DPVs of HRP/NPG/GCE bioelectrode in deaerated PBS (50 mM, pH 7.0) containing 100 μM OPD and 0.1 mM H_2_O_2_ with different sulfide concentrations; (**B**) The inhibition rate of sulfide versus the concentration of sulfide.

The electrochemical detection experiments of the HRP/NPG/GCE bioelectrode were investigated as increasing sulfide concentration using DPV technique in deaerated PBS (50 mM, pH 7.0) with the presence of 100 μM OPD and 0.1 mM H_2_O_2_. Before sulfide detection, 100 μM OPD with 0.1 mM H_2_O_2_ were added to deaerated PBS (50 mM, pH 7.0) in a three-electrode cell with the HRP/NPG/GCE bioelectrode as a working electrode to get the original current (I_0_). As shown in Fig. 3A, with the increasing of sulfide concentration, the oxidation peak current density (I_1_) of the HRP/NPG/GCE bioelectrode decreased. The inhibition rate (I,%) was calculated using the following equation^26^,^27^: 
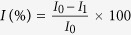
. Moreover, the inhibition rate (I, %) was proportional to sulfide concentration in the range of 0.1–40 μM, with a correlation coefficient of 0.964 as shown in Fig. 3B. Additionally, the HRP/NPG/GCE bioelectrode exhibited a high sensitivity of 1720 μA mM^−1^ cm^−2^ and a low detection limit of 0.027 μM. Notably, the HRP/NPG/GCE bioelectrode exhibited much higher sensitivity than previous reported inhibitive biosensors as shown in Table 2, which was attributed to the competitive inhibition on HRP by sulfide with OPD as a substrate. These results indicated that the HRP/NPG/GCE bioelectrode showed higher sensitivity, wider linear range, and lower detection limit than most inhibitive biosensors for sulfide detection reported in previous works (Table 2).

**Figure 4:**
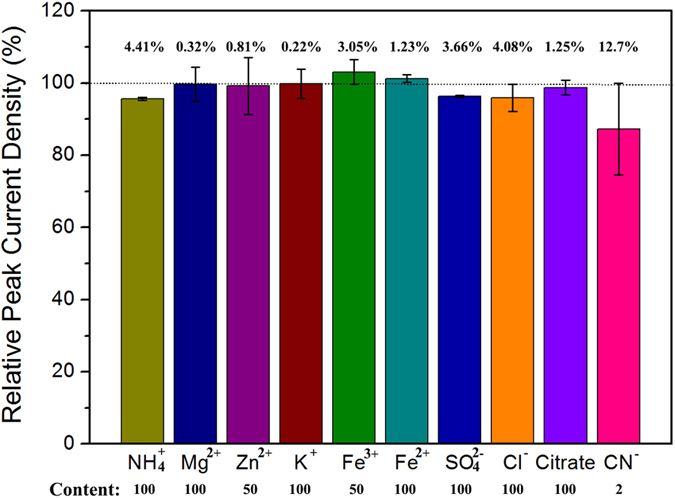
Effects of interferents on HRP/NPG/GCE bioelectrode. (Content is the ratio of interferents to sulfide)

Selectivity is a critical characteristic for an inhibitive biosensor in practical application. The effect of possible interferents on sulfide detection was studied. During this study, NH_4_^+^ (1 mM), Mg^2+^ (1 mM), Zn^2+^ (0.5 mM), K^+^ (1 mM), Fe^3+^ (0.5 mM), Fe^2+^ (1 mM), SO_4_^2−^ (1 mM), Cl^−^ (1 mM), citrate (1 mM), and CN^−^ (20 μM) were added respectively in deaerated PBS (50 mM, pH 7.0) containing 10 μM sulfide 100 μM OPD with 0.1 mM H_2_O_2_ to detected the anti-interference ability of the HRP/NPG/GCE bioelectrode. As shown in Fig. 4, negligible changes in current signal (4.41%, 0.32%, 0.81%, 0.22%, 3.05%, 1.23%, 3.66%, 4.08%, and 1.25%) were observed after the addition of NH_4_^+^, Mg^2+^, Zn^2+^, K^+^, Fe^3+^, Fe^2+^, SO_4_^2−^, Cl^−^, and citrate, respectively. This result indicated that the presence of these interferents exhibited barely interference on sulfide detection. While, cyanide (CN^−^) had an obvious interference on sulfide detection with a relative peek current density of 12.7% (Fig. 4) owing to the inhibition on HRP by cyanide^28^. Hence, cyanide should be removed for wastewater sample containing cyanide before the detection of sulfide using this bioelectrode. Overall, the HRP/NPG/GCE bioelectrode showed a strong anti-interference capability during the determination of sulfide.

The reversibility of enzyme activity inhibition is vital important for the reusability of an inhibitive biosensor. The recoverability of enzyme activity was tested by detecting the current response of the HRP/NPG/GCE bioelectrode towards OPD (100 μM) before and after sulfide detection. After the addition of 10 μM sulfide, the current response of the HRP/NPG/GCE bioelectrode was decreased. In contrast, after simply washing for 60 s using DPV technique in deaerated PBS (50 mM, pH 7.0), the current density of the HRP/NPG/GCE bioelectrode towards OPD could recover to 94% of its original current response. During the competitive inhibition process, sulfide would replace OPD on active sites of HRP, resulting in a decreased HRP activity. Sulfide combined with HRP by non-covalent bond, which could be removed by simply washing the HRP/NPG/GCE bioelectrode using DPV technique in deaerated PBS (50 mM, pH 7.0). After removal of sulfide, the active sites of HRP were free, that resulting in the recovery of enzyme activity. This result clearly demonstrated that the sulfide inhibition on HRP activity was reversible for the HRP/NPG/GCE bioelectrode.

Reproducibility and stability are basic requirements for a biosensor. The reproducibility of the HRP/NPG/GCE bioelectrode was investigated at the sulfide concentration of 10 μM. Five successive measurements were recorded with a low relative standard deviation (RSD) of 3.87%, which indicated the good reproducibility of the HRP/NPG/GCE bioelectrode. The storage stability of the HRP/NPG/GCE bioelectrode was also studied by measuring current response with 12.5 μM of sulfide after preserved at 4 °C in a refrigerator. After 40 days storage, the HRP/NPG/GCE bioelectrode could keep 97% of its original current response with RSD of 0.7% for three consecutive measurements. These results indicated that the HRP/NPG/GCE bioelectrode had good reproducibility and storage stability.

The practical detection performance of the HRP/NPG/GCE bioelectrode for sulfide was tested using two water samples. Firstly, the practical performance of the HRP/NPG/GCE bioelectrode was tested by adding certain concentration of sulfide to actual sea water sample collected from Cape of Good Hope. By substituting the inhibition rate into the calibration curve, the concentration of sulfide in the sea water sample was calculated. The results were presented in Table 3, demonstrating that the HRP/NPG/GCE bioelectrode has acceptable accuracy in sulfide detection. Furthermore, the practical performance of the HRP/NPG/GCE bioelectrode was also studied using a wastewater sample supplied by wastewater treatment plant (Jining, China). The concentration of sulfide detected by the HRP/NPG/GCE bioelectrode was very consistent with the detection result of standard spectrophotometric method. And the deviation rate was only 1.93% as shown in Table 4. These results demonstrated that the HRP/NPG/GCE biosensor was reliable and efficient for the determination of sulfide in actual water samples.

## Conclusion

In summary, an inhibitive biosensor based on the sulfide inhibition towards HRP was successfully constructed for the reliable detection of sulfide. A linear relationship was obtained between the inhibition rate of peak current and the sulfide concentrations ranged in 0.1–40 μM with a high sensitivity of 1720 μA mM^−1^ cm^−2^ and a low detection limit of 0.027 μM. The competitive inhibition towards HRP with OPD as a substrate led to a higher sensitivity in sulfide detection. Notably, the recovery of HRP activity was quickly achieved by washing the HRP/NPG/GCE bioelectrode using DPV technique in deaerated PBS (50 mM, pH 7.0) for only 60 s. Additionally, the selective detection of sulfide in real wastewater sample was achieved. Due to high sensitivity, wide determination range, good anti-interference capability, and high reversibility, the HRP/NPG/GCE bioelectrode could be an ideal choice for the reliable determination of sulfide.

## Experimental

HRP (Roche11378783, grade II) used in this study was obtained from horse radish by F. Hoffmann-La Roche Ltd. (Basel, Switzerland). Sulfide (analytical grade) and OPD (chemically pure) were purchased from Sigma-Aldrich (St. Louis, MO, USA) and Sinopharm Chemical Reagent Co., Ltd. (Shanghai, China), respectively. All other chemicals used in this study were of analytical grade. All solutions were prepared with ultrapure water.

HRP solution (4 mg mL^−1^) was prepared by adding 4 mg HRP to the mixture of 200 μL dimethyl sulfoxide and 800 μL PBS (50 mM, pH 7.0). The OPD stock solution (30 mM) was prepared using absolute ethanol, and then stored in a refrigerator at 4 °C in the dark.

GCE was polished using 50 nm Al_2_O_3_ nanoparticles on a piece of chamois leather. After that, GCE was cleaned ultrasonically in the mixture of HNO_3_ and water (v/v = 1:1), and then washed with ultrapure water and absolute ethanol for 10 min, respectively. NPG samples were made by dealloying Au_50_Ag_50_ alloy foils (wt.%, purchased from Changshu Noble Metal Company, China) in concentrated nitric acid at 30 °C for 30 min. The prepared NPG was stored in ultrapure water after being washed to pH 7.0 with ultrapure water. Finally, the NPG sample in ultrapure water was covered on the surface of GCE to construct a NPG/GCE electrode. The NPG/GCE electrode was dried in a vacuum drier for 60 min before use.

HRP solution (4 mg mL^−1^, 4 μL) was deposited on the surface of the freshly prepared NPG/GCE electrode to form HRP/NPG/GCE bioelectrode. The resulted HRP/NPG/GCE bioelectrode was stored in a refrigerator at 4 ^°^C for 72 h before use.

The surface morphology of NPG/GCE electrode and HRP/NPG/GCE bioelectrode were characterized using a Nova NanoSEM 450 field emission SEM. CV and DPV were measured using a CHI 760D electrochemical workstation (CH Corp., Shanghai, China). The three-electrode system was comprised of a working electrode (HRP/NPG/GCE), a counter electrodes (1 cm × 1 cm Pt sheet), and a reference electrode (saturated calomel electrode, SCE). All potentials were referred to the SCE and all electrochemical experiments were carried out at room temperature. PBS (50 mM, pH 7.0) used as electrolyte solution was deoxygenated by bubbling for 10 min with high purity N_2_ before each electrochemical measurement. Additionally, high purity N_2_ was full of three-electrode cell throughout each experiment.

Before electrochemical detection, 6 μL H_2_O_2_ (250 mM) stock solution was added to 15 mL deaerated PBS (50 mM, pH 7.0) to form a final H_2_O_2_ concentration of 0.1 mM in a three-electrode cell. Then 50 μL OPD (30 mM) stock solution was added to this electrolyte solution with the HRP/NPG/GCE bioelectrode as working electrode to get steady state current (I_0_) using DPV technique. Finally, different concentrations of sulfide were added to inhibit the activity of HRP with corresponding reductions in current (I_1_).

During real sea water and wastewater samples analysis, sea water (200 μL) and wastewater (5 mL) were added at the same condition to form a volume of 15 mL electrolyte solution in a three-electrode cell, respectively.

## Additional Information

**How to cite this article**: Sun, H. *et al*. Amperometric inhibitive biosensor based on horseradish peroxidase-nanoporous gold for sulfide determination. *Sci. Rep*. **6**, 30905; doi: 10.1038/srep30905 (2016).

## Figures and Tables

**Table 1:** Michaelis-Menten parameters of HRP/NPG/GCE bioelectrode.

Substrates	*I*_*max*_ (μA)	*K*^*app*^ (μM)
H_2_O_2_	3.66	36.52
H_2_O_2_ + Na_2_S	2.57	25.91
OPD	5.24	18.64
OPD + Na_2_S	5.03	21.86

**Table 2:** Performances of various types of inhibitive biosensors for sulfide detection.

Electrode	Enzyme	Detection range (μM)	Sensitivity	Detection limit (μM)	Reference
Concanavalin A/HRP/precursor modified electrode	HRP	0.1–38.5	—	0.05	1
Laponite/chitosan/GCE	HRP	0.2–8	7.801 (%, μM^−1^)	—	2
Thiolate self-assembled monolayer-Au electrode	HRP	0.5–12.7	—	0.3	11
Screen printed electrode	*Coprinus cinereus* peroxidase	1.09–16.3	—	0.3	12
Nylon membrane/Clark electrode	Ascorbate oxidase	1–15 mg L^−1^ (H_2_S)	—	0.5 mg L^−1^	14
HRP/NPG/GCE	HRP	0.1–40	1720 μA mM^−1^ cm^−2^	0.027 μM	This work

**Table 3:** Determination of sulfide in sea water sample.

Sulfide concentration in sample (μM)	Measured sulfide concentration (μM)	Deviation rate (%)
12.50	12.54	1.12
20.00	19.85	0.75
23.00	22.58	1.82
25.00	25.03	0.13
30.00	30.35	1.15

**Table 4:** Determination of sulfide in wastewater sample.

Sulfide concentration measured by proposed biosensor (μM)	Sulfide concentration measured by standard spectrophotometric method (μM)	Deviation rate (%)
49.23	50.20	1.93
